# Representation of Gender and Postgraduate Experience Among Professional Medical Society Boards in Japan

**DOI:** 10.1001/jamanetworkopen.2022.47548

**Published:** 2022-12-19

**Authors:** Takashi Watari, Ashwin Gupta, Hitomi Kataoka

**Affiliations:** 1 Shimane University Hospital, General Medicine Center, Shimane, Japan; 2 Medicine Service, Veterans Affairs Ann Arbor Healthcare System, Ann Arbor, Michigan; 3 Department of Medicine, University of Michigan Medical School, Ann Arbor; 4 Diversity and Inclusion Center, Okayama University, Okayama, Japan

## Abstract

This cross-sectional study investigates the gender ratio and postgraduate years of experience of Japanese professional medical society boards of directors.

## Introduction

The number of women physicians is increasing worldwide, including in Japan.^1,2,3^ Recognizing the prevalence of bias is essential in creating equitable systems that allow all physicians to achieve successful careers in academia. However, according to a previous study,^3^ although 22% of physicians in Japan are women, they account for only 4.7% of all professors in Japanese medical schools. Additionally, seniority bias in academia is common, especially in countries where age holds important cultural meaning. Studies^3,4^ have identified age discrimination based on seniority as a cause of decline in women professors over the past several decades. Although there are 19 major certified professional medical societies in Japan, to our knowledge, no study has investigated the gender ratio or the postgraduate years (PGYs) of experience among the boards of directors of these societies.

## Methods

In this cross-sectional study, the names of all directors or presidents, deputy directors (vice directors), board members, and auditors were extracted from the websites of the 19 specialties certified by the Japan Medical Specialists Organization on February 1, 2022 (eAppendix in Supplement 1). We obtained data on the number, gender, and PGYs of licensed physicians within each specialty using the medical qualification verification search system provided by the Ministry of Health, Labor, and Welfare in Japan. Differences in gender ratios and PGYs among the 19 societies were then assessed. This study was conducted per the Declaration of Helsinki^5^ and the Strengthening the Reporting of Observational Studies in Epidemiology (STROBE) reporting guideline. Per Japan’s Clinical Research Act, institutional review board approval and informed consent were not necessary because the data were publicly available.

Categorical variables were presented as numbers (percentages), whereas continuous variables were presented as medians (IQRs). The Mann-Whitney *U* test was used for continuous variables in the 2-group comparisons, and the χ^2^ test was used for the categorical variables. All analyses were performed using Stata SE software, version 17.0 (StataCorp LLC). Statistical significance was set at a 2-tailed *P* < .05.

## Results

**Figure 1.**
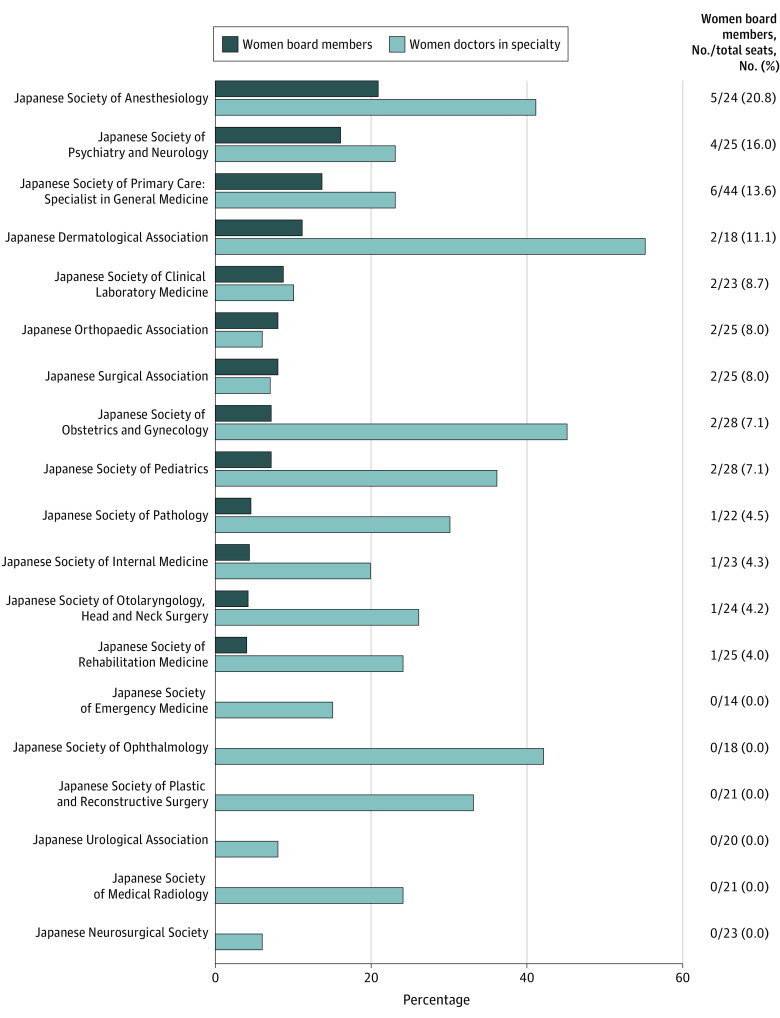
Percentage of Women Board Members Across the 19 Professional Medical Societies in Japan These data were extracted from the websites of the 19 medical specialties certified by the Japan Medical Specialties Association on February 1, 2022 (eAppendix in Supplement 1).

**Figure 2.**
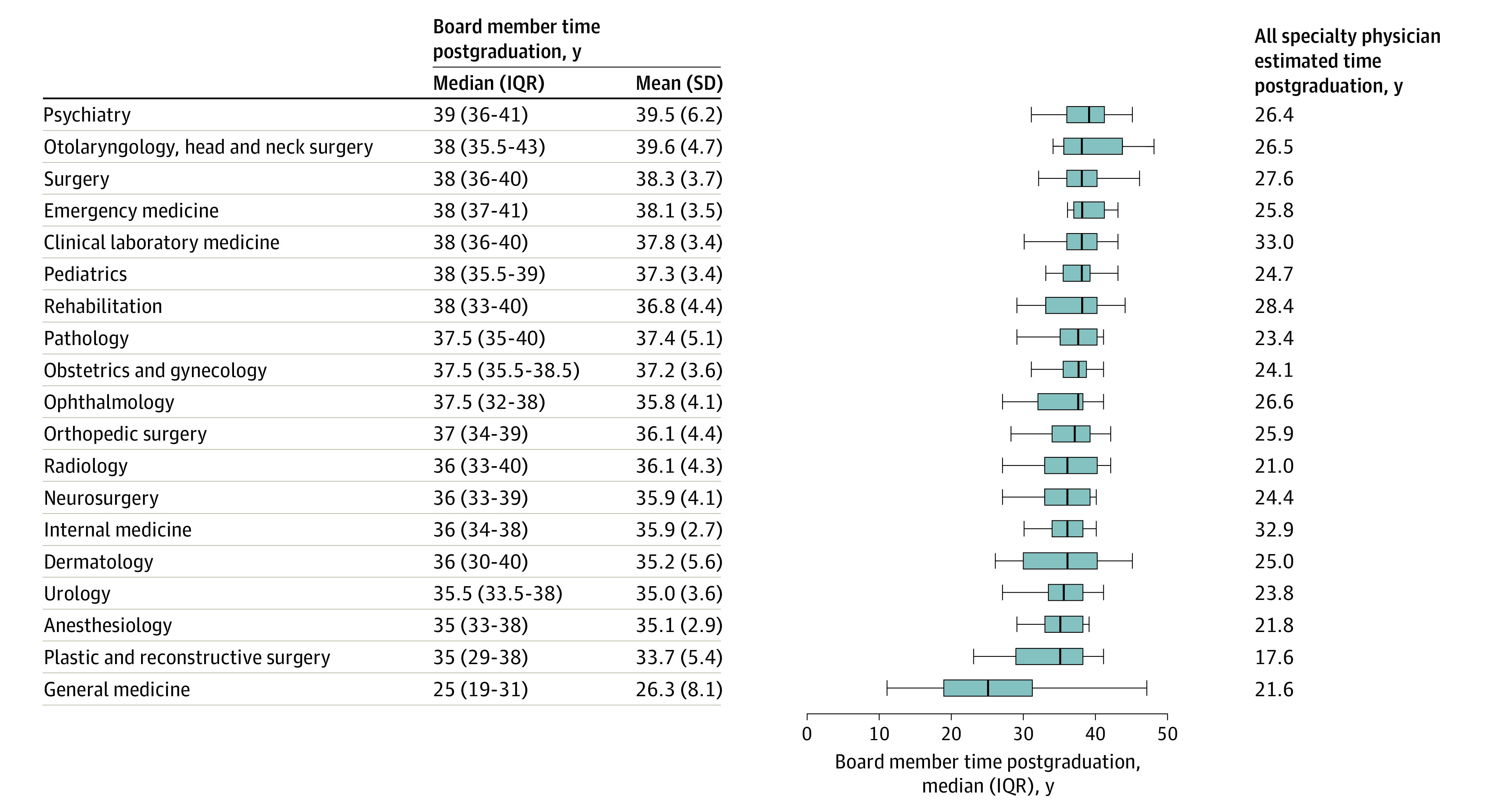
Postgraduate Years (PGYs) of the Board Members of the 19 Professional Medical Societies Statistical data are from the Ministry of Health, Labour, and Welfare and the societies’ websites. PGY-1 refers to the first year from the time of graduating from a medical school and obtaining a government-issued medical license. In Japan, after graduating from a 6-year medical school, students are required to undergo 2 years of residency education (internal medicine, surgery, obstetrics and gynecology, pediatrics, psychiatry, and elective). After that, they move on to practice in 1 of the 19 specialties. The center line for each entry represents the median; boxes, upper and lower quartile; and whiskers, maximum and minimum.

Of the 449 total board members across 19 societies, 31 (6.9%) were women. There was no difference in the median PGYs in men vs women (37 [IQR, 34-39] years vs 37 [IQR, 30-38] years; *P* = .64). All 19 society presidents were men. Anesthesiology had the highest percentage of women board members (5 of 24 [20.8%]), followed by psychiatry (4 of 25 [16.0%]) and general medicine (6 of 44 [13.6%]) (Figure 1). Surgery (2 of 25 [8.0%]) and orthopedics (2 of 25 [8.0%]) were the only societies in which the percentage of women on the board of directors was higher than that of women in total membership. General medicine board members had the lowest median years of experience vs other specialties (25 [IQR, 19-31] years vs 36 [IQR, 34-39] years, *P* < .001) (Figure 2).

## Discussion

This cross-sectional study found that women physicians are vastly underrepresented in the leadership structure of Japanese professional medical societies and constitute only 7% of board members, despite representing nearly a quarter of the workforce. Furthermore, the PGYs of board members did not reflect those of the specialty members collectively. While gender and age biases may not be the sole contributors to this phenomenon, a large body of literature suggests that these factors are pervasive in medicine and thus likely to play a role.^3,6,7^

This study has several limitations. First, the cross-sectional design of this study precludes a detailed analysis of changes in board memberships of professional medical societies over time. Second, PGYs do not necessarily reflect a physician’s age. Third, despite compelling data suggesting disparities in the makeup of professional society boards, we cannot quantify the contribution of biases to these differences. Nevertheless, this study is the first, to our knowledge, to demonstrate disparities that may be attributable to gender and seniority biases among professional medical societies in Japan. In the future, root cause analyses and their countermeasures are required to provide equal opportunities to women and early-career physicians.

## Notes

Supplement 1. **eAppendix.** Professional Medical Societies in Japan

Supplement 2.
Data Sharing Statement
