# Supplementary material for: Representation of Gender and Postgraduate Experience Among Professional Medical Society Boards in Japan
Source: JAMA Netw Open. 2022 Dec 19;5(12):e2247548. doi: 10.1001/jamanetworkopen.2022.47548 (PMC9857044; doi:10.1001/jamanetworkopen.2022.47548)
Supplement: Supplement 1. — eAppendix. Professional Medical Societies in Japan [file jamanetwopen-e2247548-s001.pdf]

Supplementary Online Content

Watari T, Gupta A, Kataoka H. Representation of gender and postgraduate experience among professional medical society boards in Japan. *JAMA Netw Open*. 2022;5(12):e2247548. doi:10.1001/jamanetworkopen.2022.47548

eAppendix. Professional Medical Societies in Japan

This supplementary material has been provided by the authors to give readers additional information about their work.

© 2022 Watari T et al. *JAMA Network Open*.

eAppendix. Professional Medical Societies in Japan

Specialty	Name of the academic medical society	URL
Internal Medicine	The Japanese Society of Internal Medicine	https://www.naika.or.jp/jsim_wp/wp-content/uploads/2021/04/yakuin_2021.pdf
Pediatrics	The Japanese Society of Pediatrics	http://www.jpeds.or.jp/modules/about/index.php?content_id=7
Dermatology	The Japanese Dermatological Association	https://www.dermatol.or.jp/modules/about/index.php?content_id=7
Psychiatry	The Japanese Society of Psychiatry and Neurology	https://www.jspn.or.jp/modules/about/index.php?content_id=30
Surgery	The Japanese Surgical Association	https://jp.jssoc.or.jp/modules/aboutus/index.php?content_id=14
Orthopedic surgery	Japanese Orthopedic Association	https://www.joa.or.jp/joa/about.html
Obstetrics and Gynecology	The Japanese Society of Obstetrics and Gynecology	https://www.jsog.or.jp/modules/about/index.php?content_id=9
Ophthalmology	The Japanese Society of Ophthalmology	https://www.nichigan.or.jp/member/about/summary/yakuin.html
Otolaryngology, Head and Neck Surgery	The Japanese Society of Otolaryngology, Head and Neck Surgery	http://www.jibika.or.jp/members/about/soshiki.html
Urology	The Japanese Urological Association	https://www.urol.or.jp/society/list/riji.html
Neurosurgery	The Japanese Neurosurgical Society	http://jns.umin.ac.jp/jns/organization
Radiology	The Japanese Society of Medical Radiology	http://www.radiology.jp/jrs_about/organization_officers.html
Anesthesiology	The Japanese Society of Anesthesiology	https://anesth.or.jp/users/common/about/officer
Pathology	The Japanese Society of Pathology	https://pathology.or.jp/jigyou/exectives.html
Clinical Laboratory Medicine	The Japanese Society of Clinical Laboratory Medicine	https://www.jslm.org/about/members/index.html
Emergency Medicine	The Japanese Society of Emergency Medicine	https://www.jaam.jp/about/shisetsu/yakuin-list.html
Plastic and Reconstructive Surgery	The Japanese Society of Plastic and Reconstructive Surgery (by Chairman/Director and Auditor)	https://jsprs.or.jp/member/officer/greeting.html
Rehabilitation	The Japanese Society of Rehabilitation Medicine	https://www.jarm.or.jp/jarm/member_yakuin.html
General Medicine	The Japanese Society of Primary Care: Specialist in General Medicine	https://www.primary-care.or.jp/about/officer.html

© 2022 Watari T et al. *JAMA Network Open*.
