# Supplementary material for: Representation of Gender and Postgraduate Experience Among Professional Medical Society Boards in Japan
Source: JAMA Netw Open. 2022 Dec 19;5(12):e2247548. doi: 10.1001/jamanetworkopen.2022.47548 (PMC9857044; doi:10.1001/jamanetworkopen.2022.47548)
Supplement: Supplement 2. — Data Sharing Statement [file jamanetwopen-e2247548-s002.pdf]

Data Sharing Statement

Watari. Representation of Gender and Postgraduate Experience Among Professional Medical Society Boards in Japan. *JAMA Netw Open*. Published December 19, 2022. doi:10.1001/jamanetworkopen.2022.47548

Data
Data available: Yes
Data types: Deidentified participant data
How to access data: The data that support the findings of this study are available from the General Medicine Center, Shimane University Hospital (E-mail. shimanegp@gmail.com), upon reasonable request.
When available: With publication

Supporting Documents
Document types: None

Additional Information
Who can access the data: Anyone requesting the data
Types of analyses: For any purpose or for a specified purpose
Mechanisms of data availability: After approval of a proposal
